# New insights on the neuroendocrine control of puberty and seasonal breeding in female sheep

**DOI:** 10.21451/1984-3143-AR2018-0047

**Published:** 2018-08-03

**Authors:** Caroline Decourt, Massimiliano Beltramo

**Affiliations:** 1 Centre for Neuroendocrinology and Department of Anatomy, University of Otago, Dunedin 9054, New Zealand; 2 INRA, UMR85 Physiologie de la Reproduction et des Comportements, F-37380 Nouzilly, France

**Keywords:** kisspeptin, ovulation, puberty onset, sheep reproduction.

## Abstract

Timing of puberty has a great influence on animal productivity. For example, reproduction in sheep can be affected by seasonality, leading to fluctuations in availability of animal products. Therefore, optimization of birth dates would improve reproductive success in sheep. Since the discovery of the major role of kisspeptin and Kiss1R, its cognate receptor, in reproductive function, there are new opportunities for interventions. Repeated or continuous administration of native kisspeptin are able to hasten puberty and induce ovulation during breeding and non-breeding seasons of sheep. However, due to the short half-life of kisspeptin, protocols involving native kisspeptin are usually proof of concept, but not practical under field conditions. Consequently, there are efforts to develop kisspeptin analogues capable of replicating effects of repeated/continuous administration of native kisspeptin. In this review, we intended to provide a comprehensive summary of the neuroendocrine requirements for puberty onset and ovulation in adult ewes, focusing on kisspeptin, its physiological effects and responses to its analogues on reproductive function in ewes.

## Introduction

In sheep, the onset of puberty occurs when there are metabolic cues that sufficient growth has occurred and when photoperiod becomes permissive. During this period, the hypothalamus become less sensitive to the negative feedback of estradiol (E2), stimulating increased pulse frequency for both gonadotropin releasing hormone (GnRH) and luteinizing hormone (LH). This increase in GnRH/LH pulse frequency increases E2 production by growing ovarian follicles, inducing an LH surge and ovulation. The timing of puberty onset has a great influence on animal productivity. Hence, a detailed understanding of mechanisms underlying initiation of puberty represents an important target for animal reproduction management, with implications for treating disorders in humans linked to anticipated or delayed puberty.

There is a clear need to improve reproductive success in livestock to provide enough products (i.e. milk and meat) to sustain a world population expected to reach 10 billion people by 2050. As livestock reproduction can be affected by seasonality, leading to fluctuations in availability of animal products, induction of ovulation during the non-breeding season is of great importance, as well as ability to control ovulation during the breeding season.

Efforts to achieve this goal have resulted in the use of molecules that activates the hypothalamo- pituitary-gonadal axis such as synthetic GnRH agonists, extracts of the reproductive hormones from human or equine origin (e.g. human chorionic gonadotropin [hCG], human menopausal gonadotropin [hMG], and equine chorionic gonadotropin [eCG]) or synthetic steroid hormones. Specific methods applying these treatments have been developed for managing livestock reproduction. However, these treatments are not entirely satisfactory. Concerning small ruminants, GnRH agonists are used rarely or not at all. Although hCG and eCG are frequently used in reproductive management, they can induce antibodies which reduce their effectiveness. In addition, production of eCG, obtained from pregnant mares, is highly questioned by animal welfare organizations and by the European Union. Therefore, efficacious, animal welfare-friendly and cost-effective novel treatments are clearly needed to improve control of livestock reproduction.

## New players in reproductive function

Among signals converging on GnRH neurons, and therefore involved in reproductive function, kisspeptin (Kp) is a recent and exciting discovery. In 2003, Kp was identified as a potent secretagogue of GnRH, based on mutation of its cognate receptor *Kiss1R* also named GPR54 (de Roux *et al*., 2003; Funes *et al*., 2003; Seminara *et al*., 2003), or of Kp gene itself (*Kiss1;*d'Anglemont de Tassigny *et al*., 2007; Dungan *et al*., 2007), resulting in hypogonadic hypogonadism and infertility. Conversely, gain-of-function mutations of *Kiss1R* cause precocious puberty (Teles *et al*., 2008).

Kisspeptins (Kps) are a group of peptides with varying numbers of amino acids (longest forms: Kp54 in human, Kp53 in sheep, or Kp52 in rodents, and smaller forms: Kp16, Kp14, Kp13 and Kp10), all derived from a common, 145 amino acid precursor. All Kps share the identical C-terminal 10 amino acids within each species, and representing the minimum endogenous sequence that activates the Kiss1R. The Kp10 sequence is relatively similar among species even if some variations can occur, suggesting a conserved physiological function (Oakley *et al*., 2009).

The gonadotropin releasing action of Kp may be due to a direct stimulatory action upon GnRH neurons at the level of hypothalamus. In sheep, this hypothesis is supported by dramatic increases in GnRH concentrations in the cerebrospinal fluid, with a parallel rise in serum LH, after intracerebroventricular (icv) administration of Kp10 (Messager *et al*., 2005). In addition, peripheral Kp10 administration can stimulate GnRH secretion (Caraty *et al*., 2013). GnRH neurons extend complex, highly branched dendritic trees beyond the blood brain barrier (BBB) into the organum vasculosum of the lamina terminalis (OVLT; Herde *et al*., 2011). This suggest a possible additional site of action of Kp other than GnRH cell bodies, via terminals of GnRH neurons in the median eminence (ME) or OVLT.

An additional site of action at the level of pituitary has also been suggested (Richard *et al*., 2009; Gahete *et al*., 2016 for revue). In sheep, Kiss1R is present in pituitary, and LH secretion increased after addition of Kp10 to pituitary cell cultures. However, Kp10 failed to induce LH release in ewes with hypothalamo-pituirary disconnection, whereas GnRH induced a significant LH release (Smith *et al*., 2008b), questioning involvement of those receptors in LH secretion. In contrast, recent data, mostly from rodents, suggest a putative role of Kp at the level of ovary, controlling follicular development, oocyte maturation, steroidogenesis and ovulation (Hu *et al*., 2017 for revue). Similarly, in a recent study, there was enhanced *in-vitro* maturation of ovine oocytes when Kp10 was added to media supplemented with follicle-stimulating hormone (FSH), LH, and E2 (Byri *et al*., 2017).

**Figure 1:**
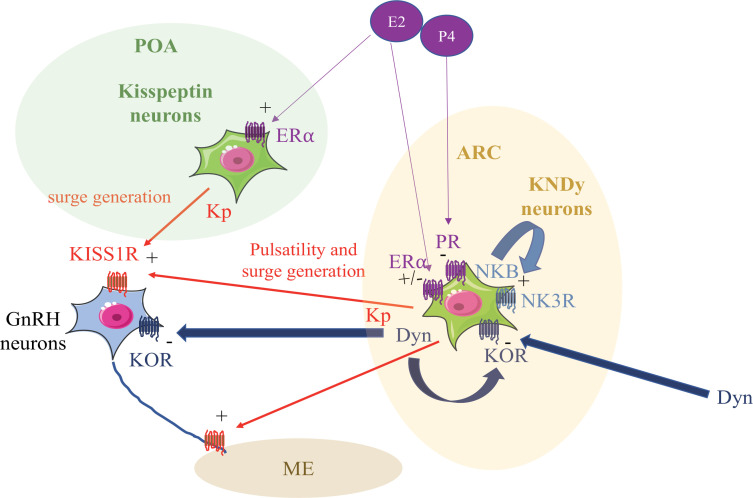
Schematic representation of Kp (Kisspeptin) and KNDy (Kisspeptin, Neurokinin B, Dynorphin) neurons regulation in adult ewes. POA (Pre Optic Area), ARC (Arcuate nucleus), ME (Median Eminence), GnRH (Gonadotropin Releasing Hormone), NKB (Neurokinin B), Dyn (Dynorphin), E2 (17 β Estradiol), P4 (progesterone), KISS1R (Kp Receptor), NK3R (NKB Receptor), KOR (Dyn Receptor), ERα (Estrogen Receptor α), PR (Progesteron Receptor).

In the hypothalamus, two distinct populations of neurons expressed Kps, the anteroventral periventricular or preoptic area (AVPV or POA) according to species, and the arcuate nucleus (ARC). These two populations are in close contact with GnRH cells (Kinoshita *et al*., 2005; Clarkson and Herbison, 2006) or their dendrons (Herde *et al*., 2011). A subpopulation of Kp neurons in the ARC have been described as co-expressing neurokinin B (NKB) and dynorphin (Dyn; Goodman *et al*., 2007) and were named KNDy neurons (Fig. 1).

NKB is also implicated in onset of puberty because mutation of *NKB* or its receptor (*NK3R*) blocked pubertal development in human (Topaloglu *et al*., 2009). In sheep, an agonist of NKBR, senktide, stimulated LH release (Nestor *et al*., 2012) whereas an antagonist of NKBR supressed GnRH/LH pulses (Clarke *et al*., 2018). In the presence of an NKBR antagonist, continuous Kp10 infusion restored GnRH/LH pulses, suggesting that Kp action is downstream of NKB signalling (Clarke *et al*., 2018). In addition, GnRH neurons do not express NK3R (Amstalden *et al*., 2010). Conversely, KNDy neurons express NK3R (Billings *et al*., 2010). These data supported the assertion that NKB acts in an autocrine/paracrine manner, indirectly influencing GnRH secretion.

Dyn, another co-expressed neuropeptide in the arcuate KNDy neurons, is an endogenous opioid peptide that selectively binds the k-opioid receptor (KOR). KOR is expressed in GnRH and KNDy neurons in ewes (Weems *et al*., 2016). There is strong evidence that Dyn tone terminates each GnRH pulse and limits amount of GnRH released during the secretory phase of the pulse (Goodman *et al*., 1995). Dyn has been implicated as a potential mediator of progesterone negative feedback effect on pulsatile GnRH secretion in ewes (Foradori *et al*., 2005) and prepubertal lambs (Lopez *et al*., 2016). However, whether this effect was due to Dyn secreted by KNDy neurons itself or by other populations, remains to be determined.

Corroborating the hypothesis of opposing effects of Dyn *vs*. Kp/NKB, Dyn expression is higher during the early follicular phase, whereas Kp/ NKB expression peak during the surge (Fergani *et al*., 2017). Based on these data, it has been suggested that KNDy neurons of the ARC nucleus could be the GnRH pulse generator.

Another recently discovered neuropeptide, GnIH (Gonadotropin-inhibitory hormone), may have a role in physiological control of reproduction, due to its inhibitory effect on GnRH release in quails (Tsutsui *et al*., 2000). However, effects of its mammalian ortholog, RFamide-related-peptide (RFRP), on GnRH/ gonadotropin secretion, is less evident. The *Rfrp* gene encodes RFRP-1, -2, and -3 peptides, but only RFRP-1 and RFRP-3 are functional peptides, with RFRP-1 stimulating prolactin secretion, and RFRP-3 modulating gonadotropin secretion. Its receptor, GPR147, was expressed in 15-33% of murine GnRH neurons (Rizwan *et al*., 2012) and in a subpopulation of Kp neurons in AVPV (5-16%) and ARC (25%; Poling *et al*., 2013). However, pubertal timing was not altered in *GPR147* KO mice (Leon *et al*., 2014) and the action of RFRP-3 on gonadotropin secretion seemed to be highly dependent on species, photoperiod, age, sex, and stage of cycle (Henningsen *et al*., 2016). It is noteworthy that RFRP-3 is sometimes inhibitory and sometimes stimulatory on LH secretion. In addition, Kp may act on GPR147, based on affinity of Kp10 for GPR147 (Roumeas *et al*., 2015).

In sheep, data were inconsistent, with an apparent inhibitory effect on LH pulse amplitude, total LH secretion, and the estrogen-induced LH surge after continuous iv infusion of RFRP-3 in ovariectomized ewes (Clarke *et al*., 2008), and a reduction of LH pulsatility during the follicular phase in intact ewes (Clarke *et al*., 2012). However, there is no association, either positive or negative, between endogenous RFRP- 3 in portal blood and LH in peripheral blood (Smith *et al*., 2012). Similarly, others reported no effects (Decourt *et al*., 2016a). Further work will be necessary to establish the role, if any, of RFRP-3 in controlling sheep reproduction.

## Regulation of steroids

GnRH neurons do not express estrogen receptor alpha (ERα Herbison and Pape, 2001) but are under estrogen positive and negative feedback. Kp neurons represent the link underlying feedback effects of steroids on GnRH secretion. The majority of Kp neurons express ERα (∼90%; Smith *et al*., 2005a, b; Franceschini *et al*., 2006), but also androgen receptor (∼65%; Smith *et al*., 2005b), and progesterone receptor (∼86%; Smith *et al*., 2007).

The ARC and AVPV populations of Kp neurons respond to sex steroids but in an opposite manner (Smith *et al*., 2005a, b). In rodents, it was proposed that Kp neurons in the AVPV integrate E2 positive feedback and therefore would be involved in LH surge generation, whereas KNDy neurons in the ARC integrate E2 negative feedback and consequently are involved in LH pulsatility.

In prebubertal lambs, Kp immunoreactive cells of the ARC region increase after ovariectomy (Nestor *et al*., 2012). Because the ovary is the main source of estrogen, this implies a negative effect of E2 on this Kp population. These data are consistent with the hypothesis that puberty is the result of a reduction in steroid negative feedback, leading to an increase in Kp secretion in ARC in prepubertal ewes. However, the recent discovery that ERα mRNA expression on Kp ARC neurons increase approaching puberty does not explain this escape (Bedenbaugh *et al*., 2018). In postpubertal ewes, E2 enhanced Kp expression in POA and concentrations were high during the late follicular phase compared to the luteal phase (Smith *et al*., 2009a). Moreover, C-Fos was induced in POA during GnRH/LH surge (Hoffman *et al*., 2011). Collectively, these data clearly demonstrated the positive feedback of E2 on Kp neurons located in POA. However, the role of ARC NKDy neurons in integrating E2 feedback is more complex, at least in ewes. Kiss1 expression in the ARC is elevated during the late follicular phase compared to the luteal phase (Estrada *et al*., 2006; Smith *et al*., 2009a), and there is C-Fos induction in Kp neurons in ARC during GnRH/LH surge (Merkley *et al*., 2012). Conversely, it was suggested (Hoffman *et al*., 2011) that ARC neurons should integrate both negative and positive E2 feedback and therefore be involved in GnRH/LH surge generation. However, caution should be exercised when making conclusions, due to potential species differences.

## Kisspeptin and puberty

**Figure 2:**
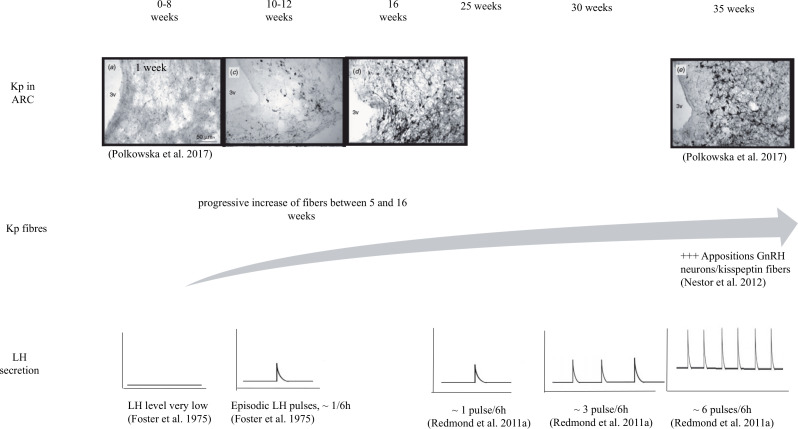
Evolution of Kp (Kisspeptin) expression in ARC (Arcuate nucleus) and LH (Luteinizing Hormone) secretion in peripheral blood, from birth to puberty onset in female lambs.

The role of Kps in reproductive function has been suggested to start early in life. Kp and its receptor are present from embryonic day 13.5 in mice (Kumar *et al*., 2014). In sheep, perturbations by administration of testosterone propionate (TP) from 30 to 58 day of gestation (GD) reduced Kiss1 mRNA expression and decreased serum LH concentrations in GD59 fetuses. Cessation of maternal TP exposure restored normal endocrine secretion after 2 week. However, even after treatment cessation, differences emerged in gene expression of GnRH, estrogen receptor-β, and Kiss1R in GD75 fetuses, suggesting that normal HPG development was disrupted (Roselli *et al*., 2016). However, no changes in Kp-ir cell numbers in the POA and ARC were observed at the adult stage in a similar study (Cheng *et al*., 2010). It remains to be determined whether changes in gene expression persist in older animals and ultimately affects timing of puberty and/or alters adult fertility. During early stages of juvenile development, the number of Kiss1-expressing cells increase in both POA and ARC. This increase in the POA was unrelated to changes in the frequency of episodic LH release. However the increase in the ARC is associated with an acceleration of pulsatile LH release during maturation of the reproductive neuroendocrine axis in ovariectomized and E2-replaced lambs (Redmond *et al*., 2011a). In addition, number of immunoreactive Kp fibers in the ARC and ME increase gradually from 5 to 16 week of age (Polkowska *et al*., 2017), concomitant with increases in plasma LH concentrations and pulse frequency (Foster *et al*., 1975; Fig. 2).

Experiments have been performed to mimic patterns of Kp release occurring during puberty using repeated Kp administration to advance puberty onset. Icv administration of 1 nmol of Kp10 every 12 h from postnatal days 26 to 31 clearly advanced onset of puberty in female rats (Navarro *et al*., 2004). In addition, repeated injections of Kp10 sustain LH and FSH pulsatility in prepubertal cattle (Ezzat Ahmed *et al*., 2009) and LH pulsatility in lambs (Redmond *et al*., 2011b). In prepubertal (28 week) Suffolk ewes, intravenous injections of 20 µg Kp10 every hour for 24 h stimulated LH pulsatility and induced an LH surge and ovulation. However, luteal activity was of short duration, with a rapid decrease in progesterone concentrations within 2 days after its initial rise, and no change in timing of puberty onset (Redmond *et al*., 2011b). Perhaps after termination of Kp treatment, spontaneous LH release was insufficient to support normal luteal function and the reproductive axis at this age is not sufficiently mature to establish regular cycles. Negative energy balance or energy excess have profound impacts on the Kp system (Manfredi-Lozano *et al*., 2018). Therefore, altering metabolic level may change the pattern of Kp secretion. This was attempted in prepubertal Tibetan ewes by supplementing either concentrates or minerals. Kiss-1, Kiss1R and ERα mRNA expression were higher in the AVPV of animals receiving concentrates and to a lesser extent in those receiving mineral supplementation compared to those eating only oat hay (Jing *et al*., 2017). In addition, follicular development was enhanced in supplemented prepubertal animals. This study supported the hypothesis that Kiss1/Kiss1R system was modulated by feed intake, and that reproductive performance was improved by this treatment.

Conversely, a study was performed to inhibit reproduction by blocking puberty onset by acting at the level of Kp. Male lambs (8 wk) were imminized against Kiss1 on weeks 0, 3 and 6 of the experiment. This treatment induced a strong anti-Kiss1 antibody titer and suppressed gonadal function and sexual behaviour. Therefore, it could be consider using *Kiss1* as a novel target for developing an immunocastration vaccine in sheep (Han *et al*., 2015).

## Impact of seasonality on kisspeptin system

In adult ewes, Kiss1 mRNA expression in ARC is higher during the breeding season compared to the non-breeding season (Wagner *et al*., 2008), with number of Kp neurons following a similar trend (Smith *et al*., 2007) suggesting that melatonin secretion influences Kiss1 expression. This effect is likely indirect, as Kp neurons do not express melatonin receptors (Li *et al*., 2011).

In addition, the inhibitory effect of E2 on Kiss1 expression in ARC is greater during the non-breeding season compared to the breeding season (Smith *et al*., 2008a). These data provide evidence that a seasonal change in estrogen sensitivity occurs at the level of Kp neurons in the ARC, leading to the switch from breeding to non-breeding seasons. In contrast, Kiss1 mARN expression in POA did not differ between breeding and non-breeding seasons and did not seem to be influenced by estrogen (Smith *et al*., 2008a). Therefore, in ewes, Kp neurons of the POA are implicated only in a positive feedback inducing an LH surge, but not in control of seasonality.

Kp induces a larger GnRH and LH increase during the non-breeding season compared to the luteal phase of the cycle (Smith *et al*., 2009b; Li *et al*., 2012). Perhaps lower pulsatility that occurs during the non- breeding season allows accumulation of a larger releasable pool of GnRH and LH compared to the luteal phase. Kiss1R expression on GnRH cells was greater during the non-breeding season than in luteal phase (Li *et al*., 2012) suggesting that low Kp concentrations during the non-breeding season induced a greater Kiss1R expression compared to the luteal phase.

Altogether, these data suggest that an increase in Kiss1R expression on GnRH neurons and the greater releasable pool of GnRH/LH contribute to the higher response of Kp in terms of GnRH/LH release during the nonbreeding season. This situation would reflect the ability of HPG to respond to an increase in GnRH pulsatility during the transition to the breeding season.

The sensitivity of HPG to Kp varies not only across seasons but also during the cycle and was correlated with Kiss1 mRNA expression. Indeed, LH response to Kp was greater during the late follicular phase in humans (Dhillo *et al*., 2007), sheep (Smith *et al*., 2009b) and rats (Roa *et al*., 2006).

## Modulation of the kisspeptin system to induce ovulation in sheep

Given the involvement of Kp in the control of reproduction in sexually mature animals, manipulation of the HPG axis using Kp treatment to promote ovulation have been attempted. However, the short half- life of this peptide (30 min for hKp54 and 1 min for hKp10 in human blood (Dhillo *et al*., 2005; Chan *et al*., 2011) requires repeated injections or continuous administration to obtain a sustained gonadotropin release. Studies conducted in human were mainly performed using Kp54, whereas for domestic animals, Kp10 represents a better compromise between efficacy and cost.

During the non-breeding season, repeated injections of Kp10 sustain LH and FSH pulse frequency in adult ewes (Caraty *et al*., 2007). However, this stimulation was insufficient to induce an LH surge. Conversely, infusion of Kp10 for 48 h (12.4 nmol/h) induced ovulation in 80% of treated animals, compared to less than 20% of control animals. A later study indicated that during the non-breeding season, a minimum of 24 h of infusion was necessary to obtain at least an ovulatory rate >75% (Sebert *et al*., 2010). During the breeding season, 8 h of Kp10 infusion (0.48 µmol/h) administered 30 h after withdrawal of a progesterone priming period, induced a preovulatory LH surge followed by ovulation (Caraty *et al*., 2007).

Although there is potential to induce ovulation with Kp10 treatment, these protocols are impractical in the field. To overcome this problem, Kp10 analogues with improved pharmacological features were developed (Table 1).

**Table 1:** Summary of effects observed on gonadotropin and/or steroid secretion and/or ovulation after kisspeptin-10 (Kp-10) or Kp-10 analog (FT080, Compound 17 or C6) administration in ewes. LH (Luteinizing Hormone), FSH (Follicle-Stimulating Hormone), E2 (17 β Estradiol), iv (intravenous), im (intramuscular).

Molecule	Ewes status	Dose and route of administration	Effect on gonadotropin and/or steroid secretion and/or ovulation	Reference
**Kp-10**	Prepubertal (28 weeks)	20 µg/h during 24 hours, iv	Increase LH pulsatility Induce ovulation	(Redmond et al. 2011b)
**Kp-10**	Adult non cyclic	6 nmol, iv	Increase LH and FSH after each injection	(Caraty et al. 2007)
**KP-10**	Adult non cyclic	15.2 nmol/h during 24h, iv	Increase LH and E2 Induce ovulation	(Sebert et al. 2010)
**Kp-10**	Adult, follicular phase	0.48 µmol/h during 8h, iv	Induce LH surge and ovulation	(Caraty et al. 2007)
**FT080**	Adult non cyclic	0.5, 2.5 or 5 nmol/kg, iv	Short lasting increase of LH (at all doses)	(Whitlock et al. 2015)
**Compound** **17**	Adult non-cyclic	15 nmol, iv	Increase LH and FSH during approximatively 9 and 5 hours respectively	(Beltramo et al. 2015)
**C6**	Adult, follicular phase	15 nmol, im	Increase LH and FSH during approximatively 12 hours Induce ovulation	(Decourt et al. 2016)
**C6**	Adult, non-cyclic	15 nmol, im	Increase LH and FSH during approximatively 12 hours Induce ovulation	(Decourt et al. 2016)

## Treatment with analogues

FTM080, a peptidomimetic containing a Gly- Leu dipeptide isostere (4-fluorobenzoyl-Phe-Gly-Leu- Arg-Trp-NH_2_) was designed to avoid hydrolysis by metalloproteinase. This analogue has an extended half- life in murine serum compared to Kp10, with comparable binding affinity and efficacy to Kp10 *in vitro* (Tomita *et al*., 2008). Effects of intravenous injection of FTM080 (0.5, 2.5, and 5.0 nmol/kg) were evaluated in Katahdin female sheep during the non- breeding season. The increase of LH was very short in amplitude and duration compared to 0.5 nmol/kg of hKp10 (Whitlock *et al*., 2015). Despite the *in vitro* improved features of FTM080 compared to Kp10, this analogue seems have a modest activity in ewes, probably due to faster renal clearance due to its small size.

We generated a series of Kp10 analogues with improved resistance to degradation. The first compounds had an enhanced *in vitro* pharmacological profile compared to Kp10, but increase in gonadotropin secretions lasted only several hours and were insufficient to induce ovulation (Beltramo *et al*., 2015). Further modifications led to the creation of the analogue named C6. This analogue combined the introduction of a triazole peptidomimetic to reduce proteolytic degradation, incorporation of an albumin-binding motif on the N-terminal amine to delay renal clearance, and methylation of arginine to enhance proteolytic stability of Kp10 (Decourt *et al*., 2016b). The C6 effect on LH secretion was tested during the breeding season by a single intramuscular injection of 15 nmol/ewe, at 24 h after the withdrawal of a 14-days progesterone pre- treatment (intravaginal sponges containing fluogestone acetate). The treatment induced synchronized LH surges 5 h after C6 injection, followed by fertile ovulations, as demonstrated by 60% pregnancy rate and birth of full- term lambs. The same protocol was performed during the non-breeding season, resulting in a synchronized LH surge 4-6 h after C6 injection, that was followed by ovulation. This treatment also triggered estrus behaviour, with ewes standing to be bred by a ram. However, pregnancy rate (40%) was lower than in the breeding season (Decourt *et al*, 2018; Centre for Neuroendocrinology and Department of Anatomy, University of Otago, Dunedin, New Zealand; unpublished data). During the non-breeding season, ovaries are not fully ready to respond to an acute stimulation and the LH surge probably induced ovulation of immature follicles, reducing fertility. This protocol was also tested in goats during breeding and non-breeding season with similar results (Decourt *et al*, 2018; Centre for Neuroendocrinology and Department of Anatomy, University of Otago, Dunedin, New Zealand; unpublished data), and highlight the necessity to further refine the protocol to improve the pregnancy rate during the non-breeding season. Perhaps a low level constant stimulation of the gonadotropic axis in order to induce follicular growth and ovulation would be preferable to pronounced, acute stimulation.

In a preliminary study, we tested the ability of C6 to advance puberty onset in prepubertal female mice. Repeated daily injections of C6 (0.15 nmol/mouse/day), from postnatal days 26 to 30, significantly advanced puberty, with vaginal opening present in all animals by day 29 *vs*. day 32 for control, and first estrus also detected much earlier in animals receiving C6 treatment (Decourt *et al*., 2016b). These results suggest a potential interest to test this treatment in livestock species, ideally with a refinement of the protocol to avoid repeated daily injections.

Takeda Pharmaceuticals have developed a nonapeptide analog, TAK-683, based on substitution of natural L-aminoacids with D-aminoacids. As mentioned earlier, this strategy is widely used to improve biological potency of peptides by increasing resistance to enzymatic degradation, although it may decrease activity due to conformational properties alteration. In cyclic goats, intravenous administration of 35 nmol of TAK-683 during the follicular phase induced an LH surge but the stimulation of LH release induced early ovulation or atresia of follicles (Goto *et al*., 2014). During an artificial luteal phase, this analogue induced a small increase of LH pulsatility within 6 h after injection, associated with an increase in E2 concentration, and followed by a surge-like release of LH with a peak at 12.5 ± 1.0 h (Endo *et al*., 2015). During pre-synchronized follicular phase, intravenous or subcutaneous administration of 3.5 nmol of TAK- 683, 12 h after withdrawal of progestogen pretreatment, induced a LH surge in the same manner, at 4.2 +/- 0.6 h and 4.6 +/- 0.4 h after iv and sc injection respectively, with ovulations detected within 3 days after injection (Kanai *et al*., 2017). However, data on fertility after these treatments are missing, and despite its good water solubility, gelation was observed within 3 h (Nishizawa *et al*., 2016). To solve this problem, they modified the analogue and created TAK-448, with no evidence of gelation within 5 days. Approximately a one-third dose of TAK-448 had similar efficacy to that of TAK-683 in rats, but efficacy on livestock species remains to be evaluated.

## Conclusions

Kp is probably the most exiting discovery of neuropeptide implicated in reproductive function since identification of GnRH. Following this discovery, we have improved our knowledge regarding mechanisms controlling this function. Furthermore, manipulating Kp signalling may provide novel potential strategies to manage livestock reproduction by controlling ovulation in adult and modulating the time of puberty onset. However, further optimization of available analogues and of experimental procedures are still needed.
